# Knowledge, attitude, and practice towards thyroid nodules and cancer among patients: a cross-sectional study

**DOI:** 10.3389/fpubh.2023.1263758

**Published:** 2023-11-03

**Authors:** Wei Li, Jian Deng, Wei Xiong, Yangyan Zhong, Hong Cao, Guoqin Jiang

**Affiliations:** ^1^Department of Breast and Thyroid Surgery, The Second Affiliated Hospital, Hengyang Medical School, University of South China, Hengyang, Hunan, China; ^2^Department of Surgery, The Second Affiliated Hospital of Soochow University, Suzhou, Jiangsu, China

**Keywords:** thyroid nodules, thyroid cancer, knowledge, attitude, practice, patient, cross-sectional study

## Abstract

**Aim:**

This study aimed to explore the knowledge, attitude, and practice (KAP) towards thyroid nodules (TN) and thyroid cancer (TC) among patients.

**Subject and methods:**

This cross-sectional study enrolled patients with TN or TC at the Second Affiliated Hospital of the University of South China between September 2022 and February 2023. A self-administered questionnaire was developed to collect demographic information of the participants, and their knowledge, attitude and practice (KAP) towards TN and TC.

**Results:**

A total of 510 valid questionnaires were collected. Among the participants, 102 (20.00%) were male, and 197 (38.63%) had the diagnosis of TC. The knowledge, attitude and practice scores were 5.76 ± 3.09 (possible range: 0–12), 31.07 ± 2.73 (possible range: 9–45), and 18.97 ± 2.92 (possible range: 5–25), respectively. Multivariate logistic regression showed that age of above 50 years old (OR = 0.27, 95%CI: 0.12–0.64, *p* = 0.003), junior college or bachelor’s degree and above (OR = 4.97, 95%CI: 1.74–14.20, *p* = 0.003), monthly income of 5,000–10,000 CNY (OR = 2.02, 95%CI: 1.09–3.74, *p* = 0.025) and > 10,000 CNY (OR = 5.67, 95%CI: 2.49–12.94, *p* < 0.001) were independently associated with knowledge. The good knowledge (OR = 3.87, 95%CI: 1.89–7.95, *p* < 0.001), high school or technical secondary school (OR = 0.52, 95%CI: 0.30–0.88, *p* = 0.016), and monthly income of 5,000–10,000 CNY (OR = 2.02, 95%CI: 1.13–3.63, *p* = 0.018) were independently associated with practice.

**Conclusion:**

Patients demonstrated poor knowledge, moderate attitude, and proactive practice towards TN and TC.

## Introduction

Thyroid nodule (TN) constitutes a prevalent endocrine disease, manifesting as localized growths in the thyroid gland due to aberrant thyroid cell proliferation (1, 2). TN patients might experience neck pain, foreign body sensation, or throat pressure, and the TN detection occurs through nodule palpation or neck ultrasound examination (3). TNs can be classified into benign and malignant types. Approximately 90% to 95% of TNs are benign; and malignant nodules account for only 5% to 15%, the majority of which represents papillary thyroid cancer (TC) (4, 5). Global statistics from the World Health Organization (WHO) indicate that TC contributed to 586,000 cases worldwide in 2020, ranking ninth in terms of incidence (6). In China, TNs have shown an escalating trend in incidence over the past two decades, with TC emerging as one of the most rapidly increasing malignancies (7). Notably, TN and TC has also been acknowledged in China’s Medium- and Long-Term Plan for Chronic Disease Prevention and Treatment (2017–2025). To mitigate the implications of these disorders, early detection, accurate diagnosis, public education, and capacity building were proposed in the plan (8).

In recent years, the rates of TN detection and incidence have been escalating (9). Managing TN entails an initial assessment of its nature and function. Asymptomatic benign nodules usually necessitate no specialized treatment, but require regular follow-up at intervals of 6 to 12 months to avert malignancy (10). If a noteworthy expansion of nodules is noted during these follow-ups, vigilant attention should be directed towards signs, symptoms, and ultrasound indications of nodule malignancy (11). Consequently, meticulous management and sustained periodic monitoring hold particular significance for patients with benign nodules (12). Malignant TN warrants early identification and treatment through surgical intervention, postoperative I^131^ therapy, and thyroid stimulating hormone (TSH) suppression therapy (13, 14). Patient-led management are largely influenced by their comprehension of treatment options and preferences (15). Consequently, evaluation of patients’ knowledge assumes critical importance, which can offer potential to enhance disease management and prognostic outcomes.

The knowledge, attitude, and practice (KAP) survey operates on the premise that human behavioral change is a sequential progression encompassing knowledge acquisition, belief formation, and behavior establishment (16). Enhancing knowledge and consciousness of TN or TC potentially contributes to better patient prognoses and reduced mortality rates (17). Previous studies have reported varying levels of understanding related to thyroid disease among the general public and university students (17–19). Nevertheless, the KAP of TN or TC patients towards these conditions and their management remained unknown. Therefore, this study aimed to unravel the KAP towards TN and TC among the patient population.

## Methods

### Study design and participants

This cross-sectional study enrolled patients with TN or TC at the Second Affiliated Hospital of the University of South China between September 2022 and February 2023. The inclusion criteria included: (1) age ≥ 18 years and (2) patients with TN detected by ultrasound or patients with TC confirmed by pathological examination. The exclusion criteria included: (1) patients unable to cooperate with the investigation and (2) incomplete questionnaire responses. The study was approved by the Ethics Committee of the Second Affiliated Hospital, University of South China (No. 202211090906), and informed consents were obtained from all participants.

### Questionnaire

The questionnaire was designed referring to American Association of Clinical Endocrinologists, American College of Endocrinology, and Association Medici Endocrinology Medical Guidelines for Clinical Practice for the Diagnosis and Management of Thyroid Nodules (20); 2022 European Thyroid Association Guidelines for the management of pediatric thyroid nodules and differentiated thyroid carcinoma (21); Expert Consensus on Postoperative Management of Differentiated Thyroid Cancer (22); and Guidelines of Chinese Society of Clinical Oncology (CSCO) Differentiated Thyroid Cancer (23). The draft was modified by two experts. A pre-test (*n* = 46) was conducted, and the Cronbach’s α was 0.817, indicating high internal consistency.

The final questionnaire was in Chinese and included 45 items. The demographic information consisted of 17 items, including their age, gender, marital status, residence, education degree, occupation, family *per capita* monthly income, medical insurance, duration of thyroid nodules, TI-RADS classification, diagnosis of TC, duration of TC, types of TC, immediate family history of TN, immediate family history of TC and immediate family history of other neoplastic diseases (other than thyroid cancer) (Table 1). The knowledge section consisted of 12 items, and the knowledge items were scored 1 point for each correct response and 0 point for a wrong response or a response of “not sure,” with a total knowledge score ranging from 0 to 12 points (Table 2). The attitude section consisted of 10 items, and the attitude items were scored on a five-point Likert scale, ranging from very positive (5 points) to very negative (1 point). The item A1 did not show positive or negative attitude tendencies, and hence, a descriptive analysis of this question was performed, and the possible score range of attitude was 9–45 points (Table 3). The last practice section consisted of 6 items, and the practice items were scored on a five-point Likert scale, ranging from very positive (5 points) to very negative (1 point). The item P6 was to investigate the information sources of participants towards TN and TC, and hence, a descriptive analysis of this question was performed. Thus, the possible score range of practice was 5–25 points (Table 4). A knowledge, attitude, and practice score ≥ 70% of the total theoretical score was considered as “good knowledge,” “positive attitude”, and “proactive practice”.

**Table 1:** Demographic characteristics of participating patients.

Characteristics	*n* (%)	Knowledge		Attitude		Practice	
		Mean ± SD	*p*	Mean ± SD	*p*	Mean ± SD	*p*
**Total**	510	5.76 ± 3.09		31.07 ± 2.73		18.97 ± 2.92	
**Age, years**			< 0.001		0.015		< 0.001
<35	113 (22.16)	8.36 ± 2.70		31.73 ± 3.23		20.23 ± 3.43	
35–50	197 (38.63)	6.04 ± 2.72		30.84 ± 2.69		18.95 ± 2.86	
>50	200 (39.22)	4.02 ± 2.45		30.94 ± 2.40		18.27 ± 2.40	
**Sex**			0.002		0.409		0.168
Male	102 (20.00)	6.62 ± 3.57		31.27 ± 3.29		19.32 ± 3.29	
Female	408 (80.00)	5.55 ± 2.92		31.02 ± 2.57		18.88 ± 2.82	
**Marital Status**			< 0.001		0.020		< 0.001
Married	397 (77.84)	5.46 ± 2.83		30.92 ± 2.58		18.65 ± 2.70	
Other (unmarried/divorced/widowed)	113 (22.16)	6.81 ± 3.69		31.60 ± 3.16		20.09 ± 3.38	
**Residence**			< 0.001		0.030		0.259
Urban	275 (53.92)	6.61 ± 2.81		31.32 ± 2.84		19.10 ± 2.86	
Non-urban (rural/suburban)	235 (46.08)	4.77 ± 3.10		30.79 ± 2.57		18.81 ± 2.99	
**Education**			< 0.001		0.001		< 0.001
Middle school and below	187 (36.67)	3.65 ± 2.44		30.69 ± 2.39		18.12 ± 2.47	
High school/ Technical secondary school	122 (23.92)	5.39 ± 2.16		30.75 ± 2.33		18.44 ± 2.53	
Junior college/ Bachelor’s degree and above	201 (39.41)	7.96 ± 2.61		31.63 ± 3.14		20.07 ± 3.18	
**Occupation**			< 0.001		0.026		0.008
Professional and technical staff	129 (25.29)	7.44 ± 2.78		31.64 ± 3.14		19.60 ± 2.80	
Production personnels in agriculture, forestry, animal husbandry, fisheries, and water resources	79 (15.49)	3.54 ± 2.65		30.91 ± 2.54		18.41 ± 2.75	
Other	302 (59.22)	5.62 ± 2.91		30.88 ± 2.56		18.84 ± 2.98	
**Family *per capita* monthly income, CNY**			< 0.001		< 0.001		< 0.001
< 5,000	347 (68.04)	4.92 ± 2.79		30.84 ± 2.52		18.33 ± 2.59	
5,000–10,000	111 (21.76)	7.01 ± 2.69		31.19 ± 2.86		19.87 ± 3.10	
>10,000	52 (10.20)	8.71 ± 3.08		32.40 ± 3.41		21.25 ± 2.99	
**Medical insurance**			0.027		0.022		0.974
Yes	502 (98.43)	5.80 ± 3.07		31.11 ± 2.73		18.97 ± 2.91	
No	8 (1.57)	3.38 ± 3.70		28.88 ± 1.89		19.00 ± 3.96	
**Duration of thyroid nodules**			0.017		0.648		< 0.001
< 6 months	73 (14.72)	6.38 ± 3.01		31.19 ± 2.51		19.00 ± 2.84	
[6 months, 1 year]	109 (21.98)	5.03 ± 2.52		31.36 ± 2.30		18.11 ± 2.32	
[1–3 years]	207 (41.73)	5.88 ± 3.07		30.95 ± 2.71		18.95 ± 2.90	
≥ 3 years	107 (21.57)	6.03 ± 3.58		31.08 ± 3.32		20.00 ± 3.30	
Missing	14 (2.74)						
**TI-RADS classification**			< 0.001		0.011		0.157
TI-RADS 0	19 (3.73)	8.53 ± 3.60		32.32 ± 3.96		20.63 ± 3.47	
TI-RADS 1	6 (1.18)	6.00 ± 4.34		31.17 ± 3.66		18.83 ± 6.15	
TI-RADS 2	21 (4.12)	7.67 ± 3.26		32.14 ± 3.15		19.76 ± 2.72	
TI-RADS 3	218 (42.75)	5.31 ± 3.14		30.65 ± 2.66		18.69 ± 2.94	
TI-RADS 4a	175 (34.31)	5.63 ± 2.64		31.48 ± 2.51		18.85 ± 2.54	
TI-RADS 4b	39 (7.65)	6.23 ± 2.87		31.05 ± 2.56		19.38 ± 2.55	
TI-RADS 4c	8 (1.57)	7.00 ± 2.83		29.50 ± 2.20		19.13 ± 2.90	
TI-RADS 5	8 (1.57)	6.13 ± 2.59		30.75 ± 2.82		19.75 ± 3.24	
Not sure	16 (3.14)	5.44 ± 4.02		30.50 ± 2.90		19.50 ± 4.62	
**Diagnosis of thyroid cancer**			0.534		0.140		0.448
No	313 (61.37)	5.69 ± 3.35		30.93 ± 2.93		19.04 ± 3.27	
Yes	197 (38.63)	5.87 ± 2.62		31.30 ± 2.37		18.84 ± 2.25	
**Duration of thyroid cancer**			0.403		0.487		0.036
< 6 months	57 (36.08)	6.23 ± 2.95		31.46 ± 2.49		19.04 ± 2.41	
[6 months, 1 year]	47 (29.75)	5.40 ± 2.31		31.60 ± 2.54		18.06 ± 1.72	
[1–3 years]	45 (28.48)	6.18 ± 2.39		30.84 ± 2.41		18.67 ± 2.25	
≥ 3 years	9 (5.70)	5.89 ± 3.59		31.22 ± 2.17		20.00 ± 2.06	
Missing	352						
**Types of thyroid cancer**			0.725		0.023		0.077
Papillary carcinoma of the thyroid	182 (92.39)	5.93 ± 2.63		31.26 ± 2.31		18.83 ± 2.26	
Follicular carcinoma of the thyroid	8 (4.06)	5.00 ± 1.85		30.25 ± 2.76		17.88 ± 1.13	
Undifferentiated carcinoma of the thyroid	0	-		-		-	
Medullary carcinoma of the thyroid	1 (0.51)	5.00		31.00		17.00	
Not sure	6 (3.05)	5.33 ± 3.44		34.00 ± 2.37		20.83 ± 2.32	
Missing	313						
**Immediate family history of thyroid nodules**			0.019		0.284		0.844
Yes	102 (20.00)	6.25 ± 2.91		31.44 ± 3.01		18.82 ± 2.80	
No	372 (72.94)	5.74 ± 3.06		31.00 ± 2.58		19.01 ± 2.90	
Unclear	36 (7.06)	4.58 ± 3.58		30.78 ± 3.36		18.92 ± 3.52	
**Immediate family history of thyroid cancer**			< 0.001		0.574		0.734
Yes	33 (6.47)	6.58 ± 2.54		30.94 ± 2.18		18.97 ± 2.70	
No	448 (87.84)	5.83 ± 3.04		31.12 ± 2.75		18.99 ± 2.91	
Unclear	29 (5.69)	3.72 ± 3.61		30.59 ± 3.08		18.55 ± 3.44	
**Immediate family history of other neoplastic diseases (other than thyroid cancer)**			0.001		0.056		0.120
Yes	29 (5.69)	7.59 ± 2.93		32.24 ± 2.94		19.66 ± 3.39	
No	409 (80.20)	5.74 ± 2.88		30.99 ± 2.62		18.84 ± 2.80	
Unclear	72 (14.12)	5.14 ± 3.93		31.11 ± 3.16		19.43 ± 3.32	

**Table 2:** Knowledge.

Items	Correct, *n* (%)			*p*
	Total	With thyroid cancer	Without thyroid cancer*	
K1. Most thyroid nodules are benign, and malignant nodules only account for about 5% of thyroid nodules.	286 (56.08)	96 (48.73)	190 (60.70)	0.008
K2. All patients with thyroid nodules should undergo a neck ultrasound examination.	351 (68.82)	148 (75.13)	203 (64.86)	0.015
K3. All patients with thyroid nodules should undergo ultrasound-guided fine-needle aspiration biopsy.	102 (20.00)	22 (11.17)	80 (25.56)	< 0.001
K4. The management for thyroid nodules shall be determined according to individual conditions, and follow-up is recommended for benign thyroid nodules.	289 (56.67)	105 (53.30)	184 (58.79)	0.223
K5. Patients with thyroid nodules with normal thyroid function require levothyroxine (L-T4) therapy.	99 (19.41)	26 (13.20)	73 (23.32)	0.005
K6. The thyroid nodules suspected of malignancy by fine-needle aspiration biopsy should be considered for surgical treatment.	369 (72.35)	152 (77.16)	217 (69.33)	0.054
K7. Surgery is the main treatment for thyroid cancer.	356 (69.80)	153 (77.66)	203 (64.86)	0.002
K8. Patients with subtotal or total thyroidectomy should take thyroxine tablets for the rest of their lives.	222 (43.53)	102 (51.78)	120 (38.34)	0.003
K9. The available surgical methods for thyroid cancer mainly include open surgery, endoscopic surgery, and ablative surgery.	255 (50.00)	101 (51.27)	154 (49.20)	0.649
K10. Parathyroid dysfunction may occur after surgery for differentiated thyroid cancer, most of which are temporary, and a few are permanent.	202 (39.61)	68 (34.5)	134 (42.81)	0.062
K11. There is a risk of recurrence after thyroid cancer surgery, and iodine 131 therapy is recommended for patients with a high risk of recurrence.	212 (41.57)	95 (48.22)	117 (37.38)	0.016
K12. The prognosis of thyroid cancer is related to pathological type, age, tumor size, etc., among which papillary cancer has the best prognosis and undifferentiated cancer has the worst prognosis.	195 (38.24)	88 (44.67)	107 (34.19)	0.018

*Participants who self-reported that they had not been diagnosed or were unclear if they had been diagnosed with TC were considered to have no thyroid cancer.

**Table 3:** Attitude.

Items	Strongly agree, *n* (%)	Agree, *n* (%)	Neutral, *n* (%)	Disagree, *n* (%)	Strongly disagree, *n* (%)
A1. You are worried after your diagnosis of thyroid disease.	67 (13.14)	204 (40.00)	197 (38.63)	33 (6.47)	9 (1.76)
A2. Thyroid nodules are very common, not a major problem, and do not need to deal with it.	2 (0.39)	44 (8.63)	134 (26.27)	295 (57.84)	35 (6.86)
A3. Even if the thyroid nodule is benign at present, it should be excised as soon as possible to prevent malignant change in the future.	37 (7.25)	120 (23.53)	164 (32.16)	180 (35.29)	9 (1.76)
A4. There is overtreatment for benign thyroid nodules at present.	13 (2.55)	68 (13.33)	219 (42.94)	195 (38.24)	15 (2.94)
A5. There is overtreatment for thyroid cancer at present.	6 (1.18)	36 (7.06)	208 (40.78)	242 (47.45)	18 (3.53)
A6. The public should be encouraged to take the initiative to screen thyroid nodules and thyroid cancer.	102 (20.00)	303 (59.41)	84 (16.47)	18 (3.53)	3 (0.59)
A7. Although the prognosis of thyroid cancer is good, surgery should be carried out as soon as possible after diagnosis.	84 (16.47)	331 (64.90)	76 (14.90)	17 (3.33)	2 (0.39)
A8. Although the prognosis of thyroid cancer is good, there is also a risk of death.	57 (11.18)	344 (67.45)	68 (13.33)	35 (6.86)	6 (1.18)
A9. After thyroidectomy, there was no need to take medicine for a long time anymore.	8 (1.57)	51 (10.00)	83 (16.27)	335 (65.69)	33 (6.47)
A10. The prognosis of thyroid cancer is good, so a regular postoperative follow-up is not needed.	6 (1.18)	27 (5.29)	79 (15.49)	345 (67.65)	53 (10.39)

**Table 4:** Practice.

Items	Very compliantly, *n* (%)	Compliantly, *n* (%)	Moderately, *n* (%)	Relatively not compliantly, *n* (%)	Not compliantly at all, *n* (%)
P1. You follow up regularly as the doctor advised.	99 (19.41)	349 (68.43)	53 (10.39)	7 (1.37)	2 (0.39)
P2. If medication is required for management, you will take medicine on time and in the dosage recommended by your doctor.	97 (19.02)	349 (68.43)	45 (8.82)	18 (3.53)	1 (0.20)
P3. After the diagnosis of thyroid nodules or thyroid cancer, you will pay attention to maintaining a good emotional state.	85 (16.67)	210 (41.18)	131 (25.69)	80 (15.69)	4 (0.78)
P4. After the diagnosis of thyroid nodules or thyroid cancer, you will pay attention to relieving your psychological pressure.	70 (13.73)	202 (39.61)	134 (26.27)	102 (20.00)	2 (0.39)
P5. You will actively learn about the management of thyroid nodules or thyroid cancer.	74 (14.51)	317 (62.16)	90 (17.65)	29 (5.69)	0

The participants were enrolled in physical examination center, outpatient clinics and wards. An online questionnaire was distributed to participants by the *Sojump* website (https://www.wjx.cn/), and participants filled out the electronic questionnaires via smartphones. Both electronic and paper questionnaires were distributed. Participants with difficulty in using electronic devices had the option to fill in a paper questionnaire instead. All participants could fill in the questionnaires only once. If participants encountered any problem in answering, members of the research group were responsible for timely interpreting the questions. After questionnaire collection, data quality checks were conducted. Questionnaires with logical errors, or repeated pattern choice were considered invalid and excluded.

### Statistical analysis

Stata 17.0 (Stata Corporation, College Station, TX, United States) was used for statistical analysis. Shapiro wilk test was used to tests for normality. Continuous data were expressed as means ± standard deviation (SD); Student’s *t*-test was used for comparison between two groups, and one-way ANOVA with Tukey’s *post hoc* test was used for continuous variables with three or more groups. The categorical data were presented as *n* (%) and compared with the chi-square test. Pearson’s correlation analysis was used to analyze the correlation between the knowledge, attitude, and practice scores. Multivariate logistic regression was performed with knowledge, attitude, and practice scores as dependent variables to explore their independent risk factors. Variables with *p* < 0.05 in univariate logistic regression were included in multivariate regression variables. Stratified analysis were performed between patients with and TC those without TC. Patients who self-reported that they had not been diagnosed or were unclear if they had been diagnosed with TC were considered “without TC.” A two-sided *p* < 0.05 was considered statistically significant.

## Results

**Figure 1:**
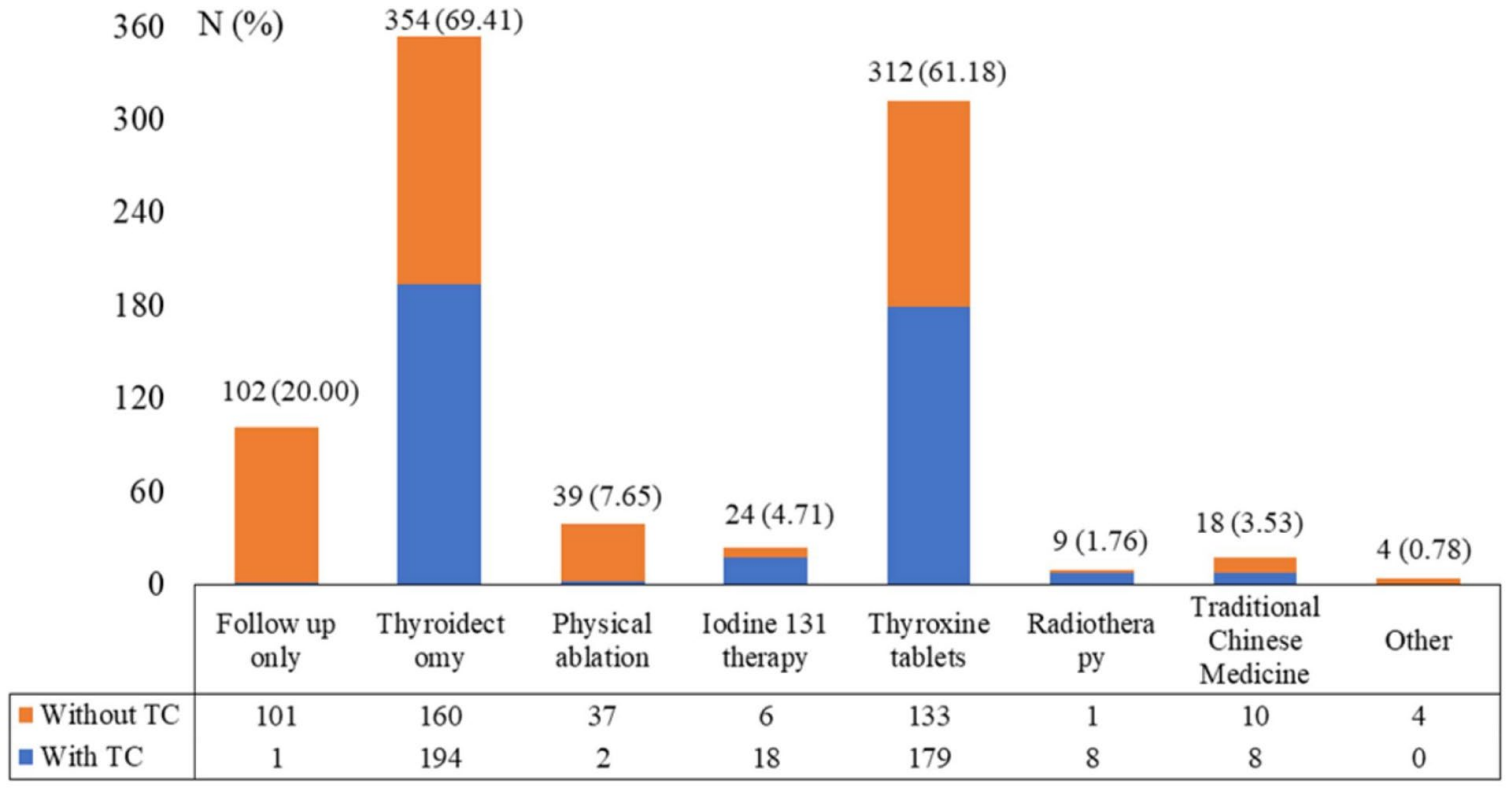
Management of the patients received for thyroid nodule and thyroid cancer.

A total of 562 questionnaires were collected, among which 52 were excluded due to logical errors or complete duplication of choices. Of the enrolled 510 participants (90.75%), 102 (20.00%) were male, 113 (22.16%) were < 35 years old, 197 (38.63%) were 35–50 years old, and 200 (39.22%) were > 50 years old. The 397 (77.84%) participants were married, 275 (53.92%) resided in urban areas, 187 (36.67%) had an education of middle school and below, 122 (23.92%) had an education of high school or technical secondary school, 129 (25.29%) were professional and technical staff, and 347 (68.04%) had a family *per capita* monthly income of <5,000 CNY. Notably, 197 (38.63%) had the diagnosis of TC confirmed by pathological examination, 102 (20.00%) had a family history of TN, and 33 (6.47%) had a family history of TC. The knowledge, attitude, and practice scores were 5.76 ± 3.09 (possible range: 0–12), 31.07 ± 2.73 (possible range: 9–45), and 18.97 ± 2.92 (possible range: 5–25), respectively. There was significant difference in knowledge scores between different age distributions, gender group, marital status, residence, education degree, occupations, family *per capita* monthly income levels, medical insurance types, duration of thyroid nodules, TI-RADS classification types, immediate family history of TN, immediate family history of TC, immediate family history of other neoplastic diseases (other than TC) (*p* < 0.05). There was significant difference in attitude scores between different age distributions, marital status, residence, education degree, occupations, family *per capita* monthly income levels, medical insurance types, TI-RADS classification types, types of TC (*p* < 0.05). There was significant difference in practice scores between different age distributions, marital status, education degree, occupations, family *per capita* monthly income levels, duration of TN, duration of TC (p < 0.05) (Table 1). The 354 (69.41%) participants had undergone thyroidectomy, 312 (61.18%) had received levothyroxine therapy, 39 (7.65%) had undergone physical ablation, and 24 (4.71%) had received I^131^ therapy (Figure 1).

There was comparable score in knowledge, attitude, and practice between participants with and without TC. Items with the lowest correct rates were that “patients with thyroid nodules with normal thyroid function require levothyroxine (L-T4) therapy” (K5) (19.41%; 26 (13.20%) among patients with TC vs. 73 (23.32%) without TC, *p* = 0.005), and “all patients with TN should undergo ultrasound-guided fine-needle aspiration biopsy” (K3) (20.00%; 22, 11.17% among patients with TC vs. 80, 25.56% without TC, *p* < 0.001). The item with the highest correct rates were K6 “the TN suspected of malignancy by fine-needle aspiration biopsy should be considered for surgical treatment” (72.35%; 152, 77.16% among patients with TC vs. 217, 69.33% without TC, *p* = 0.054), and K7 “surgery is the main treatment for TC” (69.80%; 153, 77.66% among patients with TC vs. 203, 64.86% without TC, *p* = 0.002). There were differences in correct rate of some knowledge items between those with TC and those without TC (Table 2).

More than half of the participants (271, 53.14%) agreed or strongly agreed with the disease worry, especially those with a confirmed diagnosis of TC (120, 60.91%) (A1). The majority of participants (405, 79.41%) participants agreed or strongly agreed that “the public should be encouraged to screen for TN and TC” (A6). A total of 157 (30.78%) participants agreed or strongly agreed that “even if the thyroid nodule is benign at present, it should be excised as soon as possible to prevent malignant change in the future” (A3), and 415 (81.37%) participants agreed or strongly agreed that “surgery should be carried out as soon as possible after the diagnosis of TC”(A7). However, 81 (15.88%) participants agreed or strongly agreed with the overtreatment for benign TN (A4), while 42 (8.24%) agreed or strongly agreed with the overtreatment for thyroid cancer (A5) (Table 3; Supplementary Table S1).

**Figure 2:**
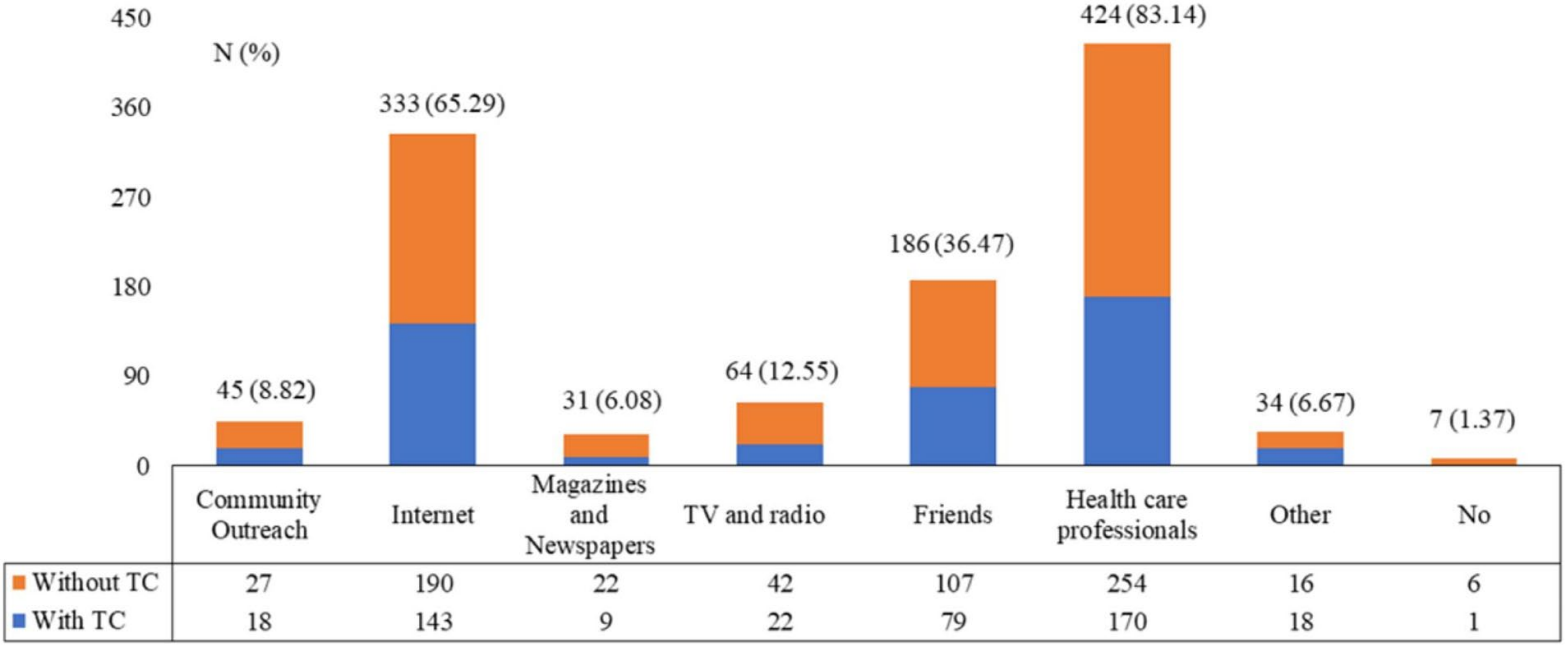
Information sources of patients towards the knowledge on thyroid nodule and thyroid cancer.

For practice, most participants claimed their compliance in follow-ups (448, 87.84%) (P1), adherence with medication regimen and administration medicine (445, 87.45%) (P2), and active learning behaviors towards the management of TN or thyroid cancer (391, 76.67%) (P5). Nearly half participants claimed that they would pay attention to maintaining a good emotional state (295, 57.85%) (P3) and relieving psychological pressure (272, 53.34%) (P4) (Table 4; Supplementary Table S2). The most common source of information was healthcare providers (424, 83.14%), followed by the Internet (333, 65.29%) and their friends (186, 36.47%) (Figure 2).

Pearson correlation analysis revealed that the knowledge scores of participants were positively correlated with their attitude scores (*r* = 0.282, *p* < 0.001) and practice scores (*r* = 0.352, *p* < 0.001), and the attitude scores were also positively correlated with their practice scores (*r* = 0.146, *p* = 0.001). Multivariate logistic regression showed that age of above 50 years old (OR = 0.27, 95%CI: 0.12–0.64, *p* = 0.003), and junior college or bachelor’s degree and above (OR = 4.97, 95%CI: 1.74–14.20, *p* = 0.003), monthly income of 5,000–10,000 CNY (OR = 2.02, 95%CI: 1.09–3.74, *p* = 0.025) and > 10,000 CNY (OR = 5.67, 95%CI: 2.49–12.94, *p* < 0.001) were independently associated with the knowledge. The knowledge score of ≥8.40 (OR = 3.87, 95%CI: 1.89–7.95, *p* < 0.001), high school or technical secondary school (OR = 0.52, 95%CI: 0.30–0.88, *p* = 0.016), and monthly income of 5,000–10,000 CNY (OR = 2.02, 95%CI: 1.13–3.63, *p* = 0.018) were independently associated with the practice. However, there was no significant association between knowledge scores and attitude scores (OR = 1.59, 95%CI: 0.99–2.54, *p* = 0.053) (Table 5).

**Table 5:** Multivariate logistic regression.

Variables	OR (95% CI)	*p*
**Knowledge**		
Age		
<35	Ref.	
35–50	0.50 (0.24, 1.01)	0.052
>50	0.27 (0.12, 0.64)	0.003
Sex		
Male	Ref.	
Female	0.94 (0.51, 1.74)	0.847
Marital status		
Married	Ref.	
Other (unmarried/divorced/widowed)	1.22 (0.63, 2.37)	0.550
Residence		
Urban	Ref.	
Non-urban (rural/suburban)	1.54 (0.81, 2.93)	0.188
Education		
Middle school and below	Ref.	
High school/Technical secondary school	1.35 (0.50, 3.64)	0.556
Junior college/Bachelor’s degree and above	4.97 (1.74, 14.20)	0.003
Occupation		
Professional and technical staff	Ref.	
Production personnel in agriculture, forestry, animal husbandry, fisheries, and water resources	0.99 (0.29, 3.37)	0.985
Other	0.79 (0.44, 1.45)	0.452
Family *per capita* monthly income, CNY		
<5,000	Ref.	
5,000–10,000	2.02 (1.09, 3.74)	0.025
>10,000	5.67 (2.49, 12.94)	< 0.001
Duration of thyroid nodule		
<6 months	Ref.	
[6 months, 1 year]	0.50 (0.19, 1.27)	0.143
[1–3 years]	0.76 (0.35, 1.62)	0.472
≥3 years	0.91 (0.38, 2.17)	0.833
Diagnosis of thyroid cancer		
No	Ref.	
Yes	0.65 (0.36, 1.18)	0.159
Immediate family history of other neoplastic diseases		
No	Ref.	
Yes	1.71 (0.61, 4.76)	0.308
Unclear	1.03 (0.49, 2.17)	0.930
**Attitude**		
Knowledge score		
[0, 8.40]	Ref.	
[8.40, 12]	1.59 (0.99, 2.54)	0.053
Education		
Middle school and below	Ref.	
High school/Technical secondary school	1.16 (0.70, 1.93)	0.555
Junior college/Bachelor’s degree and above	1.23 (0.71, 2.12)	0.465
Occupation		
Professional and technical staff	Ref.	
Production personnel in agriculture, forestry, animal husbandry, fisheries, and water resources	0.80 (0.39, 1.65)	0.548
Other	0.71 (0.44, 1.14)	0.159
Immediate family history of other neoplastic diseases		
No	Ref.	
Yes	2.04 (0.92, 4.52)	0.077
Unclear	0.84 (0.50, 1.43)	0.522
**Practice**		
Knowledge score		
[0, 8.40]	Ref.	
[8.40, 12]	3.87 (1.89, 7.95)	< 0.001
Attitude score		
[9, 31.50]	Ref.	
[31.50, 45]	1.38 (0.91, 2.11)	0.131
Age		
<35	Ref.	
35–50	1.39 (0.71, 2.71)	0.335
>50	0.92 (0.45, 1.91)	0.828
Education		
Middle school and below	Ref.	
High school/Technical secondary school	0.52 (0.30, 0.88)	0.016
Junior college/Bachelor’s degree and above	0.66 (0.35, 1.26)	0.206
Family *per capita* monthly income, CNY		
<5,000	Ref.	
5,000–10,000	2.02 (1.13, 3.63)	0.018
>10,000	2.61 (0.99, 6.89)	0.053
Duration of thyroid nodule		
<6 months	Ref.	
[6 months, 1 year]	0.52 (0.25, 1.05)	0.068
[1–3 years]	0.60 (0.31, 1.15)	0.122
≥3 years	1.02 (0.48, 2.16)	0.960

## Discussion

This study revealed that the patients had insufficient knowledge, moderate attitude, and proactive practice towards TN and TC and disease management. The findings may be used to assist healthcare providers in designing educational programs for patients to improve their awareness, attitudes, compliance, and behaviors towards TN and TC and its management.

Poor knowledge of thyroid disease is common and has been reported in previous study (24). A web-based survey showed that 56% of residents in Saudi Arabia had a poor knowledge (19). Another survey of 3,722 university students also showed that the students had poor knowledge about early signs of TC and inadequate behavior and attitude towards preventive practices of TC (17). The present study showed a misunderstanding of patients to the medication for TN and TC, the indication of fine-needle aspiration biopsy, and the prognosis of TC, indicating the need of education about the need for biopsy and treatment options. Patient preference was regarded as an important factor influencing their decision-making and greatly impacted the prognosis of TC (25). Since patient preference is affected by the knowledge and attitude towards disease, raising the knowledge and attitude appears necessary for patients to choose the appropriate treatment.

The significant differences in KAP scores among various sociodemographic factors provided valuable insights into the factors shaping individuals’ perceptions and behaviors regarding TN and TC. For example, younger individuals might exhibit greater familiarity with recent medical developments due to exposure to evolving information sources. Similarly, variations in education levels could influence the extent of medical knowledge assimilated (26). Moreover, regional disparities might contribute to differences in knowledge and attitude, since urban residents possibly have better access to healthcare information and thus cultivate more positive attitude compared to rural counterparts (27). Socioeconomic factors, including income levels and medical insurance types, might affect access to healthcare services and the ability to afford prescribed treatments. Additionally, the disparities in practice scores regarding duration of TN or TC implied the need for ongoing support and guidance for individuals facing prolonged health challenges.

Individuals without TC had higher knowledge scores in recognizing the benign nature of most TNs, the limited occurrence of malignancy, and the necessity for fine-needle aspiration biopsy. These insights were consistent with public awareness efforts and educational campaigns targeted at dispelling fears and misconceptions surrounding TNs (28). However, the lower correct rates regarding ultrasound-guided fine-needle aspiration biopsy and levothyroxine (L-T4) therapy for normal thyroid function indicated potential gaps in understanding more nuanced aspects of TN management. Conversely, patients with TC exhibited more comprehensive understanding of key aspects related to thyroid disease, which was expected given their direct engagement with the disease. Their awareness of the significance of neck ultrasound examinations, surgical interventions as the primary treatment modality, the need for lifelong thyroxine therapy post-thyroidectomy, and the intricacies of recurrence and I^131^ therapy demonstrated heightened disease-specific knowledge. This awareness was likely ascribed to their direct communications with healthcare professionals, diagnostic procedures, and treatment plans (29). Therefore, targeted efforts are needed to correct misconceptions, provide accurate information, and emphasize the significance of early detection and appropriate diagnostic measures for populations without TC. While for patients with TC, it is recommended to leverage their higher baseline knowledge to engage in shared decision-making of treatment options, potential complications, and long-term management strategies.

An online study by Park et al. found that public almost all wanted to know the diagnosis results of suspicious TN immediately, and 59.5% of respondents wanted papillary thyroid microcarcinoma removed immediately (30). This study showed similar results as most participants showed their worry about TN and TC and wanted to undergo surgeries as soon as possible. A previous qualitative interview reported that participants experienced shock, anxiety, fear, and a strong need to get TC out (31). Most patients in the present study also showed disease worry and death fear towards TC. Some participants claimed that they would not pay attention to maintaining a good emotional state and relieving psychological pressure, indicating neglect of patients on their psychological state and a lack of education on psychological support. Previous studies have suggested that the long-term psychological well-being of cancer survivors is associated with their emotional response to diagnosis (32). Therefore, improvements in the practice of psychosocial pressure management are needed.

Although surgeons and endocrinologists showed concerns about the overtreatment of thyroid disease (33), this study found that most patients disagreed with the presence of overtreatment of TN. Despite that the present results showed the good practice of participants, some participants still believed that there was no need to treat TN, take medication, and follow up as suggested by doctors. This study showed that the major source of information for patients to get knowledge of TN and TC was from doctors and nurses, and thus, more efforts should be taken to promote better health education programs in the hospital. Similar to previous studies that showed the association of education, age, and income with the KAP of participants (34, 35), this study also revealed a significant association between older age, lower income, and lower education with poorer knowledge and a lower practice score. Extra public health education programs about TN and TC should be conducted, especially for the older generation with low income and education levels. Notably, no significant association between knowledge and attitude was observed. Factors such as personal experiences, cultural beliefs, and emotional responses could exert considerable influence on attitudes, irrespective of one’s knowledge levels (36, 37). Therefore, approaching patients with sensitivity to their knowledge gaps, attitudes, and emotional responses could ensure comprehensive and effective approach to managing thyroid conditions.

Given the insufficient knowledge, it was imperative for health promotion strategies to prioritize targeted education, which should reinforce the significance of public awareness of chronic illnesses. Therefore, disseminating accurate and easily comprehensible information of TN and TC becomes pivotal. Additionally, the moderate attitudes towards thyroid conditions presented a foundation for attitude enhancement. Specifically, health promotion efforts could establish support groups for patients to exchange experiences and insights (38). Facilitated by healthcare professionals, discussions within these support groups could foster positive attitudes through knowledge exchange and emotional support. Furthermore, conducting workshops on stress management, making healthy lifestyle choices, and ensuring effective medication adherence can empower patients to actively manage their well-being.

To the best of our knowledge, this study was the first time to explore the KAP of patients towards TN and TC and its management. However, there were still several limitations. Firstly, the study relied on self-reported data, and thus was susceptible to participants’ motivations. Since offline investigations might be swayed by information interpretation of researchers, the social desirability bias might occur. Secondly, it was a single-center study, so the results may not be representative of all TN and TC patients. A multicenter, nationwide, large-sample study was needed in the future.

## Conclusion

In conclusion, patients demonstrated insufficient knowledge, moderate attitude towards TN and TC, but proactive practice regarding disease management. Education are needed to improve the knowledge of patients, especially for those aged above 50 with low income.

## Data availability statement

The raw data supporting the conclusions of this article will be made available by the authors, without undue reservation.

## Ethics statement

The studies involving humans were approved by Ethics Committee of The Second Affiliated Hospital, University of South China. The studies were conducted in accordance with the local legislation and institutional requirements. The participants provided their written informed consent to participate in this study.

## Author contributions

WL: Conceptualization, Data curation, Formal analysis, Writing – original draft. JD: Conceptualization, Data curation, Writing – original draft. WX: Conceptualization, Data curation, Writing – original draft. YZ: Data curation, Writing – review & editing. HC: Conceptualization, Formal analysis, Writing – review & editing. GJ: Data curation, Formal analysis, Writing – original draft.
