# Supplementary material for: Knowledge, attitude, and practice towards thyroid nodules and cancer among patients: a cross-sectional study
Source: Front Public Health. 2023 Nov 3;11:1263758. doi: 10.3389/fpubh.2023.1263758 (PMC10654744; doi:10.3389/fpubh.2023.1263758)
Supplement: Supplementary file 1 [file Table_1.DOCX]

Table S1 Attitude of participants with or without thyroid cancer.

|  | With thyroid cancer / Without thyroid cancer*, n (%) | | | | |
| --- | --- | --- | --- | --- | --- |
|  | Strongly agree | Agree | Neutral | Disagree | Strongly disagree |
| A1. You are worried after your diagnosis of thyroid disease. | 20 (10.15) /  47 (15.02) | 100 (50.76) /  104 (33.23) | 69 (35.03) /  128 (40.89) | 8 (4.06) /  25 (7.99) | 0 /  9 (2.88) |
| A2. Thyroid nodules are very common, not a major problem, and do not need to deal with it. | 0 /  2 (0.64) | 20 (10.15) /  24 (7.67) | 62 (31.47) /  72 (23.00) | 106 (53.81) /  189 (60.38) | 9 (4.57) /  26 (8.31) |
| A3. Even if the thyroid nodule is benign at present, it should be excised as soon as possible to prevent malignant change in the future. | 9 (4.57) /  28 (8.95) | 43 (21.83) /  77 (24.60) | 73 (37.06) /  91 (29.07) | 71 (36.04) /  109 (34.82) | 1 (0.51) /  8 (2.56) |
| A4. There is overtreatment for benign thyroid nodules at present. | 3 (1.52) /  10 (3.19) | 30 (15.23) /  38 (12.14) | 103 (52.28) /  116 (37.06) | 58 (29.44) /  137 (43.77) | 3 (1.52)/  12 (3.83) |
| A5. There is overtreatment for thyroid cancer at present. | 3 (1.52) /  3 (0.96) | 10 (5.08) /  26 (8.31) | 95 (48.22) /  113 (36.10) | 85 (43.15) /  157 (50.16) | 4 (2.03) /  14 (4.47) |
| A6. The public should be encouraged to take the initiative to screen thyroid nodules and thyroid cancer. | 29 (14.72) /  73 (23.32) | 130 (65.99) /  173 (55.27) | 32 (16.24) /  52 (16.61) | 6 (3.05) /  12 (3.83) | 0 /  3 (0.96) |
| A7. Although the prognosis of thyroid cancer is good, surgery should be carried out as soon as possible after diagnosis. | 21 (10.66) /  63 (20.13) | 145 (73.60) /  186 (59.42) | 24 (12.18) /  52 (16.61) | 7 (3.55) /  10 (3.19) | 0 /  2 (0.64) |
| A8. Although the prognosis of thyroid cancer is good, there is also a risk of death. | 15 (7.61) /  42 (13.42) | 154 (78.17) /  190 (60.70) | 16 (8.12) /  52 (16.61) | 12 (6.09) /  23 (7.35) | 0 /  6 (1.92) |
| A9. After thyroidectomy, there was no need to take medicine for a long time anymore. | 2 (1.02) /  17 (8.63) | 6 (1.92) /  34 (10.86) | 22 (11.17) /  61 (19.49) | 151 (76.65) /  184 (58.79) | 5 (2.54) /  28 (8.95) |
| A10. The prognosis of thyroid cancer is good, so a regular postoperative follow-up is not needed. | 1 (0.51) /  5 (1.60) | 9 (4.57) /  18 (5.75) | 22 (11.17) /  57 (18.21) | 157 (79.70) /  188 (60.06) | 8 (4.06) /  45 (14.38) |

* Participants who self-reported that they had not been diagnosed or were unclear if they had been diagnosed with TC were considered to have no thyroid cancer.

Table S2 Practice of participants with or without thyroid cancer.

| Items | With thyroid cancer / Without thyroid cancer*, n (%) | | | | |
| --- | --- | --- | --- | --- | --- |
|  | Very compliantly, n (%) | Compliantly, n (%) | Moderately, n (%) | Not compliantly, n (%) | Not at all, n (%) |
| P1. You follow up regularly as the doctor advised. | 27 (13.71) / 72 (23.00) | 157 (79.70) / 192 (61.34) | 13 (6.60) / 40 (12.78) | 0 / 7 (2.24) | 0 / 2 (0.64) |
| P2. If medication is required for management, you take medicine on time and in the dosage recommended by your doctor. | 27 (13.71) / 70 (22.36) | 154 (78.17) / 195 (62.30) | 16 (8.12) / 29 (9.27) | 0 / 18 (5.75) | 0 / 1 (0.32) |
| P3. After the diagnosis of thyroid nodules or thyroid cancer, you will pay attention to maintaining a good emotional state. | 26 (13.20) / 59 (18.85) | 85 (43.15) / 125 (39.94) | 55 (27.92) / 76 (24.28) | 31 (15.74) / 49 (15.65) | 0 / 4 (1.28) |
| P4. After the diagnosis of thyroid nodules or thyroid cancer, you will pay attention to relieving your psychological pressure. | 19 (9.64) / 51 (16.29) | 81 (41.12) / 121 (38.66) | 56 (28.43) / 78 (24.92) | 41 (20.81) / 61 (19.49) | 0 / 2 (0.64) |
| P5. You will actively learn about the management of thyroid nodules or thyroid cancer. | 16 (8.12) / 58 (18.53) | 134 (68.02) / 183 (58.47) | 35 (17.77) / 55 (17.57) | 12 (6.09) / 17 (5.43) | 0 / 0 |

* Participants who self-reported that they had not been diagnosed or were unclear if they had been diagnosed with TC were considered to have no thyroid cancer.
